# Clinical utility gene card for: Long-QT syndrome

**DOI:** 10.1038/s41431-021-00904-y

**Published:** 2021-05-24

**Authors:** Britt M. Beckmann, Stefanie Scheiper-Welling, Arthur A. M. Wilde, Stefan Kääb, Eric Schulze-Bahr, Silke Kauferstein

**Affiliations:** 1 Department of Legal Medicine, University Hospital Frankfurt, Frankfurt am Main, Germany; 2 Department of Medicine I, University Hospital, LMU Munich, Munich, Germany; 3 Amsterdam UMC, University of Amsterdam, Heart Center, Amsterdam, The Netherlands; 4 European Reference Network for Rare and Low Prevalence Complex Diseases of the Heart (ERN GUARDHEART; http://guardheart.ern-net.eu), Amsterdam, The Netherlands; 5 Department of Clinical and Experimental Cardiology, Meibergdreef 9, 1105 AZ Amsterdam, The Netherlands; 6 German Center for Cardiovascular Research (DZHK), Partnersite Munich, Munich, Germany; 7 Institute for Genetics of Heart Diseases, University Hospital Münster, Münster, Germany

**Keywords:** Cardiovascular diseases, Genetics research

## 1. Disease characteristic

### 1.1 Name of the disease

Long-QT syndrome (LQT, LQTS, Romano-Ward syndrome, subgroups: Jervell & Lange-Nielsen syndrome, Andersen-Tawil syndrome, Timothy syndrome, Ankyrin-B syndrome, Cardiac-only Timothy syndrome, Triadin knockout syndrome).

Comment: It may be appropriate to limit the use of numbered LQTS to LQTS 1–3 and the remaining to their pathogenic basis, such as *CALM*-LQTS rather than LQT14 [1].

### 1.2 OMIM# of the disease

#192500 (LQT1, KCNQ1-LQTS)

#613688 (LQT2, KCNH2-LQTS)

#603830 (LQT3, SCN5A-LQTS)

*#600919 (LQT4, ANK2-LQTS)*

#613695 (LQT5, KCNE1-LQTS)

*#613693 (LQT6, KCNE2-LQTS)*

#170390 (LQT7), (KCNJ2-)Andersen-Tawil syndrome

#601005, 618447 (LQT8), (CACNA1C-)Timothy syndrome

*#611818 (LQT9, CAV3-LQTS)*

*#611819 (LQT10, SCN4B-LQTS)*

*#611820 (LQT11, AKAP9-LQTS)*

*#612955 (LQT12, SNTA1-LQTS)*

*#613485 (LQT13, KCNJ5-LQTS)*

#616247 (LQT14, CALM1-LQTS)

#616249 (LQT15, CALM2-LQTS)

#114183 (LQT16, CALM3-LQTS)

n.a. (LQT 17, TRDN-LQTS)

#220400 JERVELL AND LANGE-NIELSEN SYNDROME 1; JLNS1

#612347 JERVELL AND LANGE-NIELSEN SYNDROME 2; JLNS2

Legend to Table 1.2.: Table 1.2 reflects the previous entries in OMIM. As for several of the entries, there is only disputed evidence for disease causation, those entries that are now regarded as having disputed evidence were presented in italics.

### 1.3 Name of the analysed genes or DNA/chromosome segments and OMIM# of the gene(s)

LQT1: KCNQ1, 11p15.5-p.15.4; 607542

LQT2: KCNH2, 7qq36.1; 152427

LQT3: SCN5A, 3p22.2; 600163

LQT4: ANK2, 4q25-q26; 106410

LQT5: KCNE1, 21q22.12; 176261

LQT6: KCNE2, 21q22.11; 603796

LQT7:KCNJ2, 17qq2432; 600681

LQT8: CACNA1C, 12p13.33; 114205

LQT9: CAV3, 3p25.3; 601253

LQT10: SCN4B, 11q23.3; 608256

LQT11:AKAP9, 7q21.2; 604001

LQT12: SNTA1, 20q11.21; 601017

LQT13: KCNJ5, 11q24.3; 600734

LQT14: CALM1, 14q32.11; 114180

LQT15: CALM2, 2p21; 114182

LQT16: CALM3, 19q13.32; 114183

TRDN-LQTS: 6q22.31; 603283

ATS: KCNJ2, 17qq2432; 600681

JLNS1: KCNQ1, homozygous or compound heterozygous, 11p15.5-11p15.4; 607542

JLNS2: KCNE1, homozygous or compound heterozygous, 21q22.12; 176261

TS: CACNA1C, 12p13.33; 114205

#### 1.3.1 Core genes (irrespective if being tested by Sanger sequencing or next-generation sequencing) (Table 1)

**Table 1 Core genes:** [1].

Gene	Protein	HGNC ID
KCNQ1	Potassium voltage-gated channel subfamily Q member 1	6294
KCNH2	Potassium voltage-gated channel subfamily H member 2	6251
SCN5A	Sodium voltage-gated channel alpha subunit 5	10593

#### 1.3.2 Additional genes with disease gene validity (if tested by next-generation sequencing, including whole exome/genome sequencing and panel sequencing) (Table 2)

**Table 2:** Reported genes with disease validity for LQTS [1].

Gene	Protein	HGNC ID
CACNA1C	Calcium Voltage-gated channel subunit alpha 1C	1390
CALM1	Calmodulin-1	1442
CALM2	Calmodulin-2	1445
CALM3	Calmodulin-3	1449
KCNE1	Potassium voltage-gated channel subfamily E regulatory subunit 1	6240
KCNJ2	Potassium voltage-gated channel subfamily J member 2	6263
TRDN	Triadin	12261

### 1.4 Mutational spectrum

**Table 3:** Classification of genetic evidence for genes reported to be associated with LQTS based on the work of a multicentered, international clinical domain channelopathy working group [1, 4].

Gene	LQTS	Acquired LQTS	Multiorgan subtype	Frequency
CACNA1C	Moderate		Definitive (Timothy syndrome)	~1–2% [29]
CALM1	Definitive^a^			~1–2% [30]
CALM2	Definitive^a^			~1% [30]
CALM3	Definitive^a^			<1% [31]
KCNE1	Limited	Strong		<1% [32]
KCNH2	Definitive			~25–30% [33]
KCNJ2	Limited		Definitive (Andersen-Tawil syndrome)	<1% [34]
KCNQ1	Definitive			~30–35% [35]
SCN5A	Definitive			~5–10% [36]
TRDN	Strong^b^			~2% [37]

^a^If presenting in infancy or early childhood with heart block and severe QT prolongation.

^b^Presenting with negative T waves in precordial leads, and exercise-induced arrhythmias in early childhood related to homozygous or compound heterozygous frameshift mutations.

Out of the 17 genes reported to be associated with LQTS, 3 genes (*KCNQ1, KCNH2, SCN5A*) are classified having definitive evidence as a genetic cause for LQTS. In more than 90% of the positive LQTS cases a variant affecting function is found in these three core genes. In addition, *CALM1, CALM2, CALM3* and *TRDN* have a definitive or strong evidence for disease causation, but are associated with specific features. For variants affecting function in the three *CALM* genes, LQTS may present during infancy or early childhood with heart block and severe QT prolongation. At least 30% of patients with clinical clear LQTS are genotype elusive. Common variations likely contribute to phenotype in cases without a known Mendelian variant, but it is likely that there are other genetic and non-genetic factors involved [2].

Cases with *TRDN* mutations presented during early childhood with QT prolongation, negative T waves in precordial leads and exercise-induced arrhythmia related to homozygous or compound heterozygous disease-associated variants. Cases with biallelic loss-of-function variants in *TRDN* can present with either a CPVT or LQTS-like phenotype. Prolonged QTc is not always observed. In cases where it is, it can be classified as atypical LQTS but this is not always the case. The gene *CACNA1C* was reported to have a moderate evidence for disease causation in the absence of multiorgan involvement as in Timothy syndrome. The level of evidence for the gene *KCNJ2* was only limited for the cardio-specific phenotype of LQTS, whereas both genes (*CACNA1C* and *KCNJ2*) were classified to have definitive evidence for causing multiorgan syndromes (respectively: Timothy syndrome and Andersen-Tawil syndrome). Timothy syndrome might be associated with distinctive facial features, developmental delay, endocrine abnormalities and congenital heart defects besides bradycardia, QT prolongation and polymorphic arrhythmias [3]. Extracardiac manifestations of Andersen-Tawil syndrome may present as hypo-or hypercalemic episodes of paralysis (periodic paralysis) and morphological characteristics as low set ears, clinodactyly or hypertelorism. Andersen-Tawil syndrome is still classified as LQTS although prominent U waves tempted to determine a prolonged QT interval due to inclusion of the U wave [4].

Variants affecting function in the genes *AKAP9, ANK2, CAV3, KCNE2, KCNJ5, SCN4B* and *SNTA1* are classified as having disputed evidence of disease validity and were, therefore, not included in Tables 1–3 [1, 4].

The spectrum of disease-associated variants (of the loss-of-function subtypes) contains practically all types of variants affecting function (missense, nonsense, splice site, deletions and insertions). Most patients are heterozygous for a variant affecting function, but in ~5% of the cases, patients carry two disease-associated variants in the same or different genes.

### 1.5 Analytical validation

Sequencing of all coding exons and intron-exon boundaries of the eligible genes as listed above. Analysis can be performed by Sanger sequencing, (as part of a (cardio) defined gene panel) targeted next-generation sequencing or by whole exome/genome sequencing. Deletions/duplications can be identified using different methods. For instance, multiplex ligation-dependent probe amplification, quantitative PCR, etc.

Sequencing by the Sanger method is predicted to detect >99% of variants in the target regions. Sequencing of both strands (forward and reverse) is recommended. An independent analysis of a second sample of the patient is warranted.

Using NGS as the sequencing method, the sensitivity will depend on the characteristics of test, including (sequencing strategy (e.g. panel/exome/genome), enrichment method), coverage of target regions, base quality and read depth.

Classification of detected variants should be performed according to published standards (e.g. standards of the American College of Medical Genetics and Genomics (ACMG) [5] and should be used with customisation for the specific features of LQTS and its associated genes.

### 1.6 Estimated frequency of the disease

(Incidence at birth (‘birth prevalence’) or population prevalence. If known to be variable between ethnic groups, please report):

1:2,000 in the general population. It may be assumed that the prevalence is of comparable magnitude in different populations [6].

### 1.7 Diagnostic setting

**Table:** 

	Yes.	No.
A. (Differential) diagnostics	⊠	☐
B. Predictive Testing	☐	⊠
C. Risk assessment in Relatives	⊠	☐
D. Prenatal	⊠	☐

Comments:

Comment 1: Prenatal diagnosis of Long-QT syndrome is indicated in very exceptional situations only and is asked for extremely rarely.

Comment 2: Among clinically definite LQTS cases the three most frequently affected genes with a disease-causing change are *KCNQ1, KCNH2* and *SCN5A*. Most of the LQTS patients are heterozygous for a variant affecting function, but in ~5% of the cases, patients carry two variants affecting function in the same gene (compound heterozygous or homozygous), or in different genes (digenic) [7, 8]. In general, this is associated with a more severe phenotype with younger age of onset and more adverse events, suggesting a gene-dosage effect.

Comment 3: Copy number variation might be present in 3–12% of patients in core genes [9–13].

### 2. Test characteristics

**Table:** 

	genotype or disease		A: true positives	C: false negative
	present	absent	B: false positives	D: true negative
Test				
pos.	A	B	sensitivity: specificity:	A/(A+C) D/(D+B)
neg.	C	D	pos. predict. value: neg. predict. value:	A/(A+B) D/(C+D)

### 2.1 Analytical sensitivity

(proportion of positive tests if the genotype is present in the analyte)

#### 2.1.1 if tested by conventional Sanger sequencing

Close to 100% if complete Sanger sequencing and deletion/duplication (MLPA) analysis of the affected clinically important regions of each gene is performed. MLPA is indicated for *KCNQ1*, *KCNH2* and *KCNJ2*. In *SCN5A* there is insufficient evidence for CNV causing a gain-of-function.

But this nearly 100% analytical sensitivity includes variants affecting function as well as variants that are just innocent bystanders where the clinical impact has to be proven subsequently. Potential non-coding pathogenic variants in the 3 core genes may remain undetected by standard sequencing approaches, e.g. deep intronic splice variants.

#### 2.1.2 if tested by Next-generation sequencing

Analytical sensitivity for single nucleotide variants, insertions and deletions: >99% at ≥50× read depth if MLPA and bioinformatic copy number variation (CNV) analysis is included (targeted next-generation sequencing panel approach) [14].

Lack of coverage of specific target regions is a common problem of all NGS platforms. In some cases, the problem can be particularly relevant. For example, some exons of *KCNH2* are frequently not completely covered due to their high CG-rich sequence. Thus, additional analysis by Sanger sequencing of these uncovered regions is often required [15, 16]. Core genes (*KCNQ1*, *KCNH2*, *SCN5A*) including flanking splice sites should be entirely and sufficiently covered (at least 20x). Potential non-coding pathogenic variants in the 3 main genes may remain undetected by standard sequencing approaches, e.g. deep intronic splice variants.

### 2.2 Analytical specificity

(proportion of negative tests if the genotype is not present)

#### 2.2.1 if tested by conventional Sanger sequencing

Close to 100% if complete sequencing and MLPA of the affected gene is performed. But not finding a disease-associated variant rejects by no means the diagnosis LQTS in definite clinical cases as in about 30% the underlying cause or causative genes are still not known.

#### 2.2.2 if tested by Next-generation sequencing

See 2.2.1

### 2.3 Clinical sensitivity **(proportion of positive tests if the disease is present)**

The clinical sensitivity can be dependent on variable factors such as age or family history. In such cases, a general statement should be given, even if a quantification can only be made case by case.

#### 2.3.1 if tested by conventional Sanger sequencing

On average the detection rate of variants affecting function for the most frequent LQTS disease genes (*KCNQ1*, *KCNH2*, and *SCN5A*) is about 60–70% [17].

#### 2.3.2 if tested by next-generation sequencing

Extra sensitivity due to sequencing additional genes by NGS is minimally higher as each additionally tested gene mentioned in table a.1.3.1 and 1.4 increases sensitivity slightly.

### 2.4 Clinical specificity (proportion of negative tests if the disease is not present)

The clinical specificity can be dependent on variable factors such as age or family history. For instance, some patients show an incomplete phenotype, in some individuals the diagnosis is established in adulthood, because of a late onset of symptoms. These cases can likely result in a lower sensitivity. In such cases, a general statement should be given, even if a quantification can only be made case by case.

#### 2.4.1 if tested by conventional Sanger sequencing

About 95%, however, the rate of rare variants of uncertain significance (i.e. non-synonymous genetic variation) in Caucasians is about 4–8% in Non-Caucasian in the LQTS 1–3 genes [18].

#### 2.4.2 If tested by next-generation sequencing

See 2.4.1

### 2.5 Positive clinical predictive value (life time risk to develop the disease if the test is positive)

Before the age of 40 years roughly 40% of (untreated) patients with LQTS1 and LQTS2 become symptomatic. In LQTS3 this is less, but symptoms may be more severe. Phenotypic expression of the disorder is time-dependent and LQTS subjects maintain a high risk for life-threatening cardiac events after age 40 years, which seems to be less high for LQTS1 [19, 20].

### 2.6 Negative clinical predictive value (Probability not to develop the disease if the test is negative)

(Probability not to develop the disease if the test is negative).

Assume an increased risk based on family history for a non-affected person. Allelic and locus heterogeneity may need to be considered.

Index case in that family had been tested:

If the index case in that family had been tested and a non-equivocal disease-associated variant had been found in the index patient and the non-affected proband is not a carrier of the identified disease- associated variant close to 100%. In those cases the risk remains as small as the prevalence of the disease in the general population.

Index case in that family had not been tested:

If the patient is clinically affected (prolonged QTc with or without syncope) the index patient has a chance of about 60–70% carrying a variant affecting function. But only in very rare cases there is an indication for performing LQTS genetic testing in a clinically unaffected relative when the index case has not been tested.

This could be imaginable when in an index case there is a strong clinical suspicion of LQTS and there is no DNA available or the index patient refuses genetic testing. Usually, there is no indication for genetic testing in a clinically unaffected family member with unclear genetic status of the index patient if the ECG is normal.

## 3. Clinical Utility

### 3.1 (Differential) diagnostics: The tested person is clinically affected (To be answered if in 1.9 "A" was marked)

#### 3.1.1 Can a diagnosis be made other than through a genetic test?

**Table:** 

No.	□ (continue with 3.1.4)	
Yes.	⊠	
	Clinically	⊠
	Imaging	□
	Endoscopy	□
	Biochemistry	□
	Electrophysiology	
	Other (please describe)	ECG recording
	Measurement of the QTc interval on repeated ECG recordings and typical clinical symptoms (with low sensitivity) [21].	

#### 3.1.2 Describe the burden of alternative diagnostic methods to the patient

ECGs are a non-invasive procedure with no risks and little inconvenience for the patient. But for the reason of low sensitivity and specificity the burden is psychological: uncertainty of proper diagnosis as well as appropriate clinical care: individual therapy, individual recommendations for treatment, life style adaption and individual risk stratification based on specific subtype are not possible in the absence of a genetic substrate.

#### 3.1.3 How is the cost effectiveness of alternative diagnostic methods to be judged?

As far as a disease-causing mutation is identified in the index patient, genetic testing can be offered to apparently healthy relatives within the family in order to determine if they carry the same variant affecting function and are at risk for malignant ventricular arrhythmias. If the relative carries the known disease-associated variant a prophylactic inexpensive medical treatment can be started and specific advice can be given to gene carriers (avoiding substances/drugs which might trigger arrhythmias, avoidance of genotype-specific triggers for arrhythmias, careful attendance in case of pregnancy and delivery, reproductive counselling, counselling concerning choice of profession). There is a reduction of the relative risk for developing serious cardiac events of about 65% by proper treatment (mostly with an inexpensive betablocker therapy) and the cardiac events in untreated patients on the other hand may lead to early invalidity or death in otherwise often healthy young people with putative high economic loss.

#### 3.1.4 Will disease management be influenced by the result of a genetic test?

**Table:** 

No.	□	
Yes.	⊠	
	Therapy (please describe)	Pharmaceutical treatment (usually beta-blockers) as primary and secondary prevention. In rare individual cases additional pacemaker and/or an implantable cardioverter defibrillator (ICD), and/or left cardiac sympathetic denervation (LCSD) is used. The implantation of an ICD with or without the performance of LCSD is mostly reserved for patients in which optimal non-invasive therapy and lifestyle modifications fail to protect against ventricular arrhythmias. Only in exceptional individual cases ICD implantation might be indicated for primary prevention. For more specific therapeutic recommendations please see [22].
		Pharmacotherapy might differ between genotypes. Also, life style advices are different for the different genotypes [23, 24].
	Prognosis (please describe)	In general, even in asymptomatic subjects carrying the disease associated variant, regular preventive medical check-ups to recognise disease progression early, and appropriate treatment improve prognosis. On appropriate treatment and with appropriate life style adjustments prognosis is good in the vast majority of patients and may not differ from normal [25, 26].
	Management (please describe)	Regular preventive medical consultation of carriers of a variant affecting function, adjustment or intensification of therapy if appropriate (including defibrillator implantation and/or LCSD), if necessary.
		The molecular-genetic information is very important for counselling and clinical management related to the different subtypes of the disease.

### 3.2 Predictive setting: the tested person is clinically unaffected but carries an increased risk based on family history

(To be answered if in 1.9 "B" was marked)

#### 3.2.1 Will the result of a genetic test influence lifestyle and prevention?

Regular cardiological check-ups.

Life style adjustment, avoiding event related triggers (e.g. swimming in LQTS1 and loud acoustic stimuli in LQT2) [7, 8]. Avoiding stringent competition sports (LQTS1). Avoiding QT prolonging drugs (www.crediblemeds.org; all subtypes) and avoidance of fever (especially in LQTS2) [27, 28].

Some disease associate variants have an unusually high clinical severity (e.g., KCNQ1 A341V) [9]. Also patients with compound heterozygous variants affecting function and JLNS patients are at a higher risk.

If the test result is **negative** (please describe):

Precautionary measures as described above are not needed.

#### 3.2.2 Which options in view of lifestyle and prevention does a person at-risk have if no genetic test has been done (please describe)?

Same as described above. However, the preventive measures are much better accepted and compliance is improved if a positive test result was obtained.

### 3.3 Genetic risk assessment in family members of a diseased person

(To be answered if in 1.9 ‘C’ was marked)

#### 3.3.1 Does the result of a genetic test resolve the genetic situation in that family?

If a disease-associated variant is found: yes. Otherwise, potentially affected relatives should undergo regular cardiologic evaluation.

#### 3.3.2 Can a genetic test in the index patient save genetic or other tests in family members?

No. But in case of a positive test result in the index patient, clinically asymptomatic relatives being non-carriers of the disease associate variant can be excluded from regular cardiologic follow-up.

#### 3.3.3 Does a positive genetic test result in the index patient enable a predictive test in a family member?

No. But it enables a diagnostic test in family members with a normal ECG (~40% of individuals carrying a variant affecting function).

### 3.4 Prenatal diagnosis

(To be answered if in 1.9 ‘D’ was marked)

#### 3.4.1 Does a positive genetic test result in the index patient enable a prenatal diagnosis?

Yes, but prenatal diagnostics are not actively offered.

## 4. If applicable, further consequences of testing

Please assume that the result of a genetic test has no immediate medical consequences. Is there any evidence that a genetic test is nevertheless useful for the patient or his/her relatives? (Please describe)

For every patient (clinically affected or not) there are known specific triggers for arrhythmias to be avoided (e.g. QT prolonging drugs, competitive sports, low potassium serum levels, swimming in LQTS1, sudden loud noise in LQTS2) [7, 8, 23, 24]. Thus, there should be thorough counselling concerning lifestyle modifications and choice of employment.

This work was supported by EuroGentest2 (Unit 2: ‘Genetic testing as part of health care’), a Coordination Action under FP7 (Grant Agreement Number 261469) and the European Society of Human Genetics.
